# Spatiotemporal Correlation Spectroscopy Reveals a Protective Effect of Peptide-Based GLP-1 Receptor Agonism against Lipotoxicity on Insulin Granule Dynamics in Primary Human β-Cells

**DOI:** 10.3390/pharmaceutics13091403

**Published:** 2021-09-03

**Authors:** Gianmarco Ferri, Marta Tesi, Luca Pesce, Marco Bugliani, Francesca Grano, Margherita Occhipinti, Mara Suleiman, Carmela De Luca, Lorella Marselli, Piero Marchetti, Francesco Cardarelli

**Affiliations:** 1 Laboratorio NEST-Scuola Normale Superiore, Piazza San Silvestro 12, 56127 Pisa, Italy; gianmarco.ferri@sns.it (G.F.); luca.pesce1@sns.it (L.P.); 2 Department of Clinical and Experimental Medicine, Islet Cell Laboratory, University of Pisa, 56127 Pisa, Italy; marta.tesi91@gmail.com (M.T.); m.bugliani@ao-pisa.toscana.it (M.B.); f.grano@ao-pisa.toscana.it (F.G.); m.occhipinti@ao-pisa.toscana.it (M.O.); mara.suleiman@for.unipi.it (M.S.); carmela.deluca3288@gmail.com (C.D.L.); lorella.marselli@med.unipi.it (L.M.); piero.marchetti@med.unipi.it (P.M.)

**Keywords:** pancreatic islets, β-cells, GLP-1 receptor agonism, syncollin, *i*MSD, insulin secretory granule dynamics, fluorescence

## Abstract

Glucagon-like peptide-1 receptor (GLP-1R) agonists are being used for the treatment of type 2 diabetes (T2D) and may have beneficial effects on the pancreatic β-cells. Here, we evaluated the effects of GLP-1R agonism on insulin secretory granule (ISG) dynamics in primary β-cells isolated from human islets exposed to palmitate-induced lipotoxic stress. Islets cells were exposed for 48 h to 0.5 mM palmitate (hereafter, ‘Palm’) with or without the addition of a GLP-1 agonist, namely 10 nM exendin-4 (hereafter, ‘Ex-4’). Dissociated cells were first transfected with syncollin-EGFP in order to fluorescently mark the ISGs. Then, by applying a recently established spatiotemporal correlation spectroscopy technique, the average structural (i.e., size) and dynamic (i.e., the local diffusivity and mode of motion) properties of ISGs are extracted from a calculated imaging-derived Mean Square Displacement (*i*MSD) trace. Besides defining the structural/dynamic fingerprint of ISGs in human cells for the first time, *i*MSD analysis allowed to probe fingerprint variations under selected conditions: namely, it was shown that Palm affects ISGs dynamics in response to acute glucose stimulation by abolishing the ISGs mobilization typically imparted by glucose and, concomitantly, by reducing the extent of ISGs active/directed intracellular movement. By contrast, co-treatment with Ex-4 normalizes ISG dynamics, i.e., re-establish ISG mobilization and ability to perform active transport in response to glucose stimulation. These observations were correlated with standard glucose-stimulated insulin secretion (GSIS), which resulted in being reduced in cells exposed to Palm but preserved in cells concomitantly exposed to 10 nM Ex-4. Our data support the idea that GLP-1R agonism may exert its beneficial effect on human β-cells under metabolic stress by maintaining ISGs’ proper intracellular dynamics.

## 1. Introduction

Pancreatic β-cell dysfunction, determined by the interplay of genetic and acquired factors, has a major role in the development and progression of type 2 diabetes (T2D) [1,2]. In this regard, increased concentrations of certain fatty acids (palmitate in particular) have been shown to induce a lipotoxic effect and impair β-cell function, survival and even proliferation [3,4,5]. Among the several pharmacological treatments for the therapy of diabetes, GLP-1 receptor (GLP-1R) agonists are being used, with a favorable benefit/risk ratio [6,7]. These compounds, in fact, have several beneficial actions, including possible protection of β-cells against metabolic stresses [8] or against death by increasing autophagic flux and restoring lysosomal function [9]. In spite of much work conducted to investigate the cellular and subcellular mechanisms of lipotoxicity and, in turn, of the protective effect of GLP-1R agonists [10,11,12], the overall scenario is still not fully understood. Worthy of mention, in the last decade, the insulin secretory granule (ISG) attracted growing interest as an essential subcellular node for signaling in the β-cell, and not merely as a (insulin) container/carrier [13]. Indeed, modifications of ISG structural (e.g., size) and dynamic (e.g., diffusivity) properties are being found as hallmarks of pancreatic β-cells dysfunction in many circumstances. For instance, hypercholesterolemia was associated with an overall increase in granule size accompanied by impaired granule trafficking [14]; type-1 diabetes (T1D) onset was demonstrated to be accompanied by, among others, an ISG change in structural and functional properties (through fusion with lysosomes) [15]. In spite of such general interest and preliminary indications, however, rapid and robust measurement of ISGs’ structural and dynamic properties in living β-cells remains a challenging task. Two limit strategies are currently available, but each with specific limitations: on the one hand, Transmission Electron Microscopy (TEM) provides ultrastructural details, but at the expenses of information on dynamics (and, for what concerns specifically ISGs, prone to artifacts [16]); on the other hand, fluorescence-based optical microscopy allows to study ISG dynamics in a living matter [17,18,19,20,21], but with (i) limited or null access to structural information, and (ii) limited efficacy if applied to a three-dimensional environment where many of the objects are packed closer than the resolution limit of the optical setup, as in the case of labeled ISGs. In this context, some of us recently introduced an algorithm of spatiotemporal fluctuation analysis that simultaneously extracts the average structural (i.e., size) and dynamic (i.e., diffusivity, anomalous coefficient) properties of diffusing objects directly from the standard time-series of optical microscopy images with no need to extract single-object trajectories [22]. The corresponding experimental protocol consists of a few steps. First, imaging of the region of interest is performed at high temporal resolution. Then, average spatial-temporal correlation functions are calculated from the stack of images. Finally, by Gaussian fitting of the series of correlation functions, the average ‘diffusion law’ is obtained directly from imaging, in the form of the so-called imaging-derived Mean Square Displacement (*i*MSD) [22]. At this point, the characteristic parameters describing ISG average structural properties (i.e., size) and dynamics (i.e., diffusion coefficient, D, and anomalous coefficient, α, at different spatiotemporal scales) can be readily extracted and used to define the fingerprint of the structure of interest. The potential of the method was already demonstrated for a variety of biological objects, ranging from molecules to nanoparticles or entire subcellular organelles/structures [22,23,24,25,26,27,28]. In particular, some of us recently validated the *i*MSD approach to the use of fluorescently labeled ISGs in a model of β-like immortalized cells, the insulinoma-1E (INS-1E) cells [26]. With this in mind, here we propose the application of the *i*MSD approach to evaluate the effect of palmitate-induced lipotoxicity (‘Palm’ treatment) and potential protection by the GLP-1 agonist Exendin-4 (‘Ex-4’ treatment) on ISGs in primary living β-cells dissociated from human pancreatic islet (dHI). To this end, cells disaggregated from human islets were transiently transfected with syncollin-EGFP in order to fluorescently mark the insulin granules. Then, the *i*MSD analysis was used as a fast and robust tool to define the structural/dynamic fingerprint of ISGs under the conditions of interest. In brief, it was shown that Palm affects ISGs dynamics in response to acute glucose stimulation by abolishing the ISGs mobilization typically imparted by glucose and, concomitantly, by reducing the extent of ISGs active/directed intracellular movement. By contrast, co-treatment with Ex-4 normalizes ISG dynamics, i.e., re-establish ISG mobilization and ability to perform active transport in response to glucose. These effects are correlated with standard glucose-stimulated insulin secretion (GSIS), which resulted in being significantly reduced in cells exposed to Palm but preserved in cells concomitantly exposed to 10 nM Ex-4. Present results allow postulating a beneficial effect of GLP-1R agonism exerted at the level of intracellular insulin granules, in particular by maintaining their dynamic properties at physiological levels.

## 2. Materials and Methods

### 2.1. Human Pancreatic Islets

Pancreata from 11 non-diabetic organ donors (age 64.9 ± 13.4; sex 8M/3F; body mass index 24.7 ± 3) were used for islet isolation, through procedures approved by the Ethics Committee of the University of Pisa. Islets were isolated by collagenase digestion followed by density gradient purification, as previously reported [29,30], and cultured at 37 °C, 5% CO_2_ atmosphere, in M199 culture medium supplemented with 10% bovine serum, 100 U/mL penicillin, 100 μg/mL streptomycin, 750 ng/mL amphotericin B and 50 μg/mL gentamicin (Sigma-Aldrich, St. Louis, MO, USA). Within 3 days from isolation, islets were exposed to 0.5 mM palmitate (Sigma-Aldrich, St. Louis, MO, USA) for 48 h with or without 10 nM of Exendin-4 (Sigma-Aldrich, St. Louis, MO, USA). Palmitate was dissolved in 90% ethanol, heated to 60 °C and 1:100 diluted to a final concentration of 0.5 mM and a molar ratio of palmitate:bovine serum albumin of 3.33, corresponding to an unbound palmitate concentration of 27 nM [31]. Control incubations contained the same concentrations of ethanol and albumin. In certain experiments, islet cells were dissociated as follows. Approximately 600 islets were suspended in calcium-free Krebs Ringer Bicarbonate solution, containing 1 mmol/L EGTA. Dispersed islet cells were obtained by adding 100 μg/mL trypsin and 8 μg/mL Dnase (Roche Diagnostics, Mannheim, Germany), at 37 °C. The samples were checked every 2 min, and the digestion was stopped by adding cold Krebs Ringer Bicarbonate solution when mostly single cells or small cell aggregates (three to five cells) were detected. Dispersed cells were washed carefully with culture medium by centrifugation at 300× *g* for 2 min and cultured on biTreat µ-Dish 35 mm, high walls, #1.5 polymer coverslip, tissue culture treated, sterilized and fluorescence microscopy suitable (Ibidi, Martinsried, Germany), previously treated with Matrigel.

### 2.2. Insulin Secretion Assays

Batches of 15 handpicked islets, previously cultured at the above-mentioned conditions, were pre-incubated at 3.3 mM glucose for 45 min, then challenged with 3.3 mM glucose for 45 min, followed by other 45 min incubation at 16.7 mM glucose [29,32]. Islet insulin content was measured after acid–alcohol extraction, as previously reported [29,32]. Insulin was quantified by a radioimmunometric assay (DIAsource ImmunoAssays S.A., Nivelles, Belgium). Insulin release was expressed as Insulin Stimulation Index (ISI), calculated as the ratio of insulin release at 16.7 mM glucose over release at 3.3 mM glucose.

### 2.3. Immunostaining

Guinea pig polyclonal antibody against total insulin (preproinsulin, proinsulin and insulin) from Abcam, ab7842 was used to perform an immunostaining experiment on dispersed HI cells. The Alexa Fluor 541-conjugated goat anti-mouse secondary antibody (LifeThermo Fisher, Waltham, MA, USA) was used to detect signals for imaging experiments.

### 2.4. Plasmid Transfection

Dispersed human islet cells were transfected using lipofectamine 2000 reagent as per manufacturer’s instructions using Optimem culture media to dilute reagents (LifeTechnologies, Thermo Fisher, Waltham, MA, USA) and cells were cultured for 48 h prior to microscopy. Syncollin-EGFP plasmid was a kind gift of Micheal Edwardson (Department of Pharmacology, University of Cambridge).

### 2.5. Fluorescence Microscopy and *i*MSD Analysis

Fluorescence measurements on dHI cells were carried out with Zeiss LSM 800 inverted confocal microscope (Jena, Germany). Images were acquired illuminating the sample with a 488 nm laser for EGFP using a 63× (N.A. 1.4) oil-immersion objective. EGFP fluorescence was collected between 500 and 600 nm with a GaAsP detector. For *i*MSD analysis, each acquisition consists of a collection of 500 frames (256 × 256 pixels) at a temporal resolution of 204 ms/frame and with a pixel size of 50 nm. The theoretical framework and main applications of *i*MSD analysis can be found in Refs. [1,2,3] for molecules diffusing within cells and in Refs. [4,5,6,7,8] for studying the motion of sub-cellular organelles/nanostructures in the cell cytoplasm. Briefly, a time-lapse series of 500 frames were analyzed using a custom script working on MATLAB (MathWorks Inc., Natick, MA, USA), which computes by Fast Fourier methods the spatiotemporal correlation function, defined as follows:

(1) $$g\left(\xi ,\eta ,\tau \right)=\frac{i\left(x,y,t\right)i\left(x+\xi ,y+\eta ,t,+t\right)}{i{\left(x,y,t\right)}^{2}}-1$$

g(ξ,η,τ) can be fitted with a standard Gaussian function:

(2) $$g\left(\xi ,\eta ,\tau \right)=g_{0}+g_{1}\left(\tau \right)\exp\left\{-\frac{{\left(\xi -\nu_{\xi }\tau \right)}^{2}+{\left(\eta -\nu_{\eta }\tau \right)}^{2}}{\sigma^{2}\left(\tau \right)}\right\}$$

whose variance σ^2^(τ) is analogous to the mean square displacement extracted directly from imaging, *i*MSD. Of particular note, the apparent particle size could be calculated using:

(3) $$Size_{app}=\sqrt{\sigma_{0}^{2}}$$

In this case $Size_{app}$ (apparent) represents the average diameter of imaged ISGs, i.e., the real size of the ISGs convolved with the instrument’s PSF. To obtain the actual average ISGs size could be used the following relation:

(4) $$Size_{act}=\frac{2}{\sqrt{2}}\sqrt{\sigma_{0}^{2}-PSF_{waist}^{2}}$$

### 2.6. Cluster Similarity Analysis

The measured dynamic parameters (i.e., the short-scale diffusion coefficient D, the *i*MSD intercept value σ_0_ and the exponent of anomalous diffusion α) of each image-stack define a data point in a three-dimensional space. Thus, the set of data points corresponding to the dynamics of a specific system is a 3D multivariate distribution of the measured values. To quantify a degree of similarity among the investigated dynamics, we calculated the statistical difference between two distributions, as described in a previous report [8].

### 2.7. ISGs Tracking Analysis

Trajectories analysis was performed using TrackMate plugin for ImageJ (Bethesda, MD, USA). LogDetector algorithm was applied to detect fluorescence spots; the Lap Tracker algorithm was used to perform tracking analysis.

## 3. Results and Discussion

### 3.1. *i*MSD-Based Structural/Dynamic Fingerprint of ISGs from Human β-Cells

**Figure 1:**
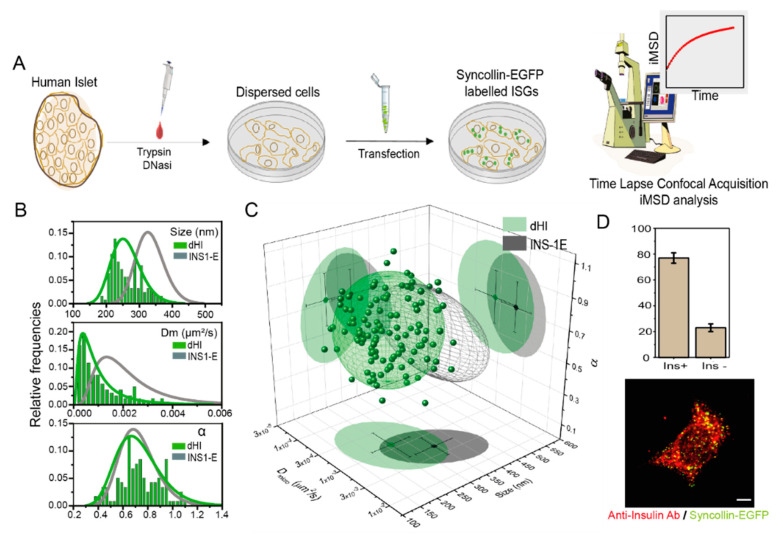
*i*MSD-based screening of ISGs structural and dynamic properties in dispersed human β-cells, as compared to immortalized INS-1E model. (**A**) Schematic representation of HI dispersion and transfection process. Forty-eight hours post-transfection, fluorescently labeled dispersed cells were observed with a confocal microscope to measure ISG structural-dynamic properties. (**B**) Distributions of the three *i*MSD relevant parameters (size, D_m_ and α) of *n* = 123 dispersed human islet cells (green curve and bars) compared with previous data retrieved from syncollin-EGFP labeled ISGs from INS-1E cells (grey curve and bars) (CIT). (**C**) Three-dimensional parametric plot showing the ‘fingerprint’ of syncollin-EGFP labeled ISGs (represented as 68% confidential ellipsoid) of human dispersed cells (green) and INS-1E (dark grey). (**D**) Exemplary image of a syncollin-EGFP (green) transfected dispersed islet cell immunostained with Alexa-541 Anti-insulin Ab (red). Scale bar: 5 μm. Column bars show the percentage of insulin-Ab positive (~75%) and Ab-negative in transfected cells (~25%) in *n* = 3 samples of dispersed human islets.

First, we sought to define the structural and dynamic fingerprint of ISGs in human-derived primary β-cells. The general workflow of our experiments is schematically represented in Figure 1a (further details about islet isolation, cell disaggregation and transfection can be found in Materials and Methods). Thus, primary human cells disaggregated from islets of Langerhans were plated and transiently transfected with syncollin-EGFP to obtain labeled ISGs suitable for fluorescence microscopy analysis. Please note that syncollin-EGFP overexpression, based on previous results on INS-1E cells [8], does not significantly alter ISG structural and dynamic properties, contrary to what was observed using alternative granule markers (e.g., Phogrin-FPs). Technically, time-lapse series of about 500 images were acquired and analyzed by the *i*MSD algorithm^1^: measured *i*MSD traces are reported, for the sake of clearness, in Figure S1. As already demonstrated [7,8], the *i*MSD algorithm can extract information on ISGs average diffusion law and, upon fitting, parameters describing their structure (i.e., their average size) and motility (i.e., the local diffusivity (D_m_) and the anomalous (α) diffusion coefficients) directly from standard imaging without the need to extract individual trajectories. Normalized distributions extracted by fitting procedures of size, D_m_, and α are plotted in Figure 1b (green histograms) and compared to data obtained from similarly-labeled ISGs in INS-1E cells (grey curve, taken from Ref. [26], Figure S1). The triplet of size, D_m_ and α values for each analyzed human-derived cell are shown in a 3D plot in Figure 1c, together with the 68% confidential ellipsoid (green), as compared to data from INS-1E cells (represented only by the 68% confidential ellipsoid, grey). A cluster similarity analysis yields a value of statistical cluster distance (SCD) of 0.536, indicating only partial superimposition of the two clusters (SCD = 0 total superimposition, SCD = 1 absence of superimposition) (Table 1). Worthy of mention, in fact, *i*MSD analysis reveals that the ISG fingerprint in human-derived cells is substantially different from that obtained in INS-1E cells. In more detail, the average size of ISGs is nearly 30% smaller in primary human-derived cells as compared to INS-1 E cells (see Table 1). Please note that this result well agrees with TEM-based estimates obtained on similar models: Rosengren and co-workers, in fact, reported a mean granule diameter from primary human β-cells (285 ± 11 nm [33]), sensibly lower than what was reported for granules in INS-1 E cells (>315 nm [34]). In addition, ISGs from human β-cells show here a decreased local diffusivity (1.2 × 10^−3^ µm^2^/s) as compared to their immortalized counterparts (2.4 × 10^−3^ µm^2^/s), although the mean values of the α anomalous coefficients are identical (~0.71). These results indicate that structural and dynamic properties of ISGs from human primary and immortalized β-cell are not identical, but these differences in the average size and local diffusivity are not surprising, also in light of the growing body of evidence supporting the idea that intracellular vesicles/organelles might be inherently altered, in terms of structural and trafficking properties, in immortalized cellular models as compared to primary cell models (for a review see [35]). Worthy of mention, in parallel, disaggregated and transfected cells were also fixed and immunostained against insulin to distinguish β-cells from non-β-cells (Figure 1d). This control experiment assures that nearly 75% of the syncollin-EGFP expressing cells analyzed are actually true β-cells in our assays, as they are positive for insulin.

### 3.2. GLP-1 Agonism Effect on Human β-Cell ISGs under Lipotoxic Stress

**Figure 2:**
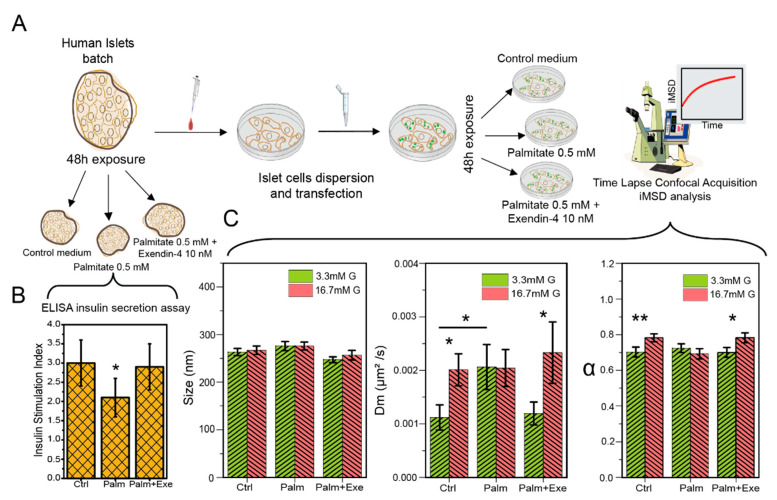
The effect of Exendin-4 on palmitate-treated dispersed human β-cells. (**A**) Workflow of the experimental procedure. Isolated human Langerhans islets were exposed for 48 h to control medium (Ctrl), 0.5 mM Palm and 0.5 mM Palm + 10 nM Exe-4 and then tested for insulin secretion. In a parallel set of experiments, HI from the same donor were dissociated and transfected with Syncollin-EGFP plasmid in order to label ISGs. Dispersed cells were exposed for 48 h to conditions described above, then fluorescently labeled ISGs’ structural and dynamic properties were evaluated in a confocal microscope experiment. (**B**) Insulin stimulation index measured with ELISA kit assay for intact islets exposed to control medium, Palm and Palm + Exe-4. (* *p* < 0.05). (**C**) *i*MSD derived parameters (size, D_m_ and α) were measured in a low (3.3 mM) glucose concentration and after stimulation with high (16.7 mM) glucose in Ctrl, Palm and Palm + Ex-4 treated cells. Data represented as Mean ± SE (* *p* < 0.05, ** *p* < 0.01—*t*-test). ‘Ctrl’: control medium; ‘Palm’: 0.5 mM palmitate; ‘Palm + Exe’: 0.5 mM palmitate + 10 nM Exendin-4.

The *i*MSD-based fingerprinting procedure can now be used as a fast and robust tool to evaluate the effect of lipotoxicity induced by Palm treatment and the possible protective effect elicited by Ex-4, as detailed in the following. To this end, the established protocol was slightly modified to include standard ELISA-based insulin secretion assays to be performed just before islet disaggregation in the three relevant conditions of control, exposure to Palm and co-exposure to Palm and Ex-4 (workflow in Figure 2a). The insulin stimulation index (ISI) of control islets (incubation for 48 h in plain M199 culture medium, see Methods for further details) was 3.0 ± 0.6. As expected, prolonged exposure to 0.5 mM palmitate caused a reduction in the ISI to 2.1 ± 0.5 (*p* < 0.05). However, the concomitant presence of 10 nM Exe-4 in the palmitate-containing medium prevented the reduction in ISI, which resulted in being 2.9 ± 0.6. Overall, these observations are in keeping with what was found in previous studies [36,37,38].

In parallel experiments, cells dissociated from islets and transfected with syncollin-EGFP were exposed to the control medium, Palm or Palm + Exe-4, and analyzed by *i*MSD. The extracted parameters are used to build ISGs fingerprints in all the relevant experimental conditions. The structural/dynamic fingerprint of control cells responds to acute glucose stimulation (passing from 3.3 to 16.7 mM) as expected based on previous data on INS-1E cells [26]. In fact, glucose does not impact the average ISG size (Figure 2c left plot, Table 2) but induces an increase in both ISG characteristic local diffusivity, D_m_ (Figure 2c middle plot, Table 2), and α coefficient (Figure 2c right plot, Table 2). Worthy of mention, the variations in both parameters observed in human cells are of the same magnitude (~2-folds increase in D_m_, ~1.15-folds increase in α) of those measured in immortalized cells by some of us [26] and others [21]. Such an effect of glucose on the average dynamic properties of ISGs is commonly interpreted as the combined result of granule mobilization (increase in diffusivity) and overall commitment to secretion by active/directed intracellular movements, presumably along with cytoskeleton components. This picture was also confirmed, in INS-1E cells, by experiments in which similar fingerprint variations (e.g., increase in α upon stimulation) were abolished by cytoskeleton disruption or cholesterol overload [26]. With this in mind, we analyzed the effect of glucose stimulation in cells exposed to 0.5 mM Palm. Please note that the average size of ISGs is not affected by Palm neither in low nor high glucose conditions (Figure 2c, left plot; Table 2). By contrast, ISGs dynamic properties are substantially altered. In particular, Palm is able to abolish the increase in both granule D_m_ and α coefficients typically induced by glucose stimulation (Figure 2c, middle-right plot; Table 2). At the same time, co-treatment with 10 nM Exe-4 is sufficient to restore granule dynamics, both in terms of D_m_ and α, to the same extent as control cells (Figure 2c, middle-right plot; Table 2), revealing a protective effect of this compound against the observed effects of lipotoxicity. We are prompted to speculate that ISGs may have a reduced propensity to perform active transport in presence of palmitate. As demonstrated elsewhere both for ISGs [26] and other organelles [27], the *i*MSD-derived α coefficient is averaged over the whole population of granules captured during imaging: as such, it reflects the sum of all single-granule contributions. This latter can be appreciated by standard analysis of single-granule trajectories (exemplary cases for the experimental conditions tested here are reported in Figure S2).

As mentioned above, these results from human-derived cells mirror what was observed in INS-1E cells in previous work [26]. In our opinion, it is interesting to note that the palmitate-induced lipotoxic effect does not modify the ISG characteristic size. In turn, this may suggest that the mechanism of action of palmitate does not imply palmitate accumulation at the ISG membrane or, in general, direct action on the ISG structure, contrary to what was observed for cholesterol, which was able to induce a ~40% increase in ISG size by direct accumulation at the granule-membrane level [14,26]. In keeping with this, it was suggested that palmitate-induced lipotoxicity in β cells might be exerted through either a mitochondria- or ER-dependent pathways [5,39], both leading to cellular stress (e.g., ROS production), inflammation and, finally, β-cell damage. Indeed, it was also postulated that the protective role of GLP-1 receptor agonists or other compounds (e.g., oleate) might be played by stimulating pro-survival and/or anti-inflammatory mechanisms [40,41].

## 4. Conclusions

In conclusion, in our experimental conditions, GLP-1R agonism shows a previously unknown beneficial effect exerted at the level of intracellular insulin granules, in particular by maintaining their dynamic properties at physiological levels. In brief, it was shown that Palm affects ISGs dynamics in response to acute glucose stimulation by abolishing the ISGs mobilization effect imparted typically by glucose and, concomitantly, by reducing the extent of granule active/directed intracellular movement. Of note, co-treatment with Exe-4 is sufficient to normalize ISG dynamics. These effects are correlated with standard glucose-stimulated insulin secretion (GSIS), which resulted in being significantly reduced in cells exposed to Palm but preserved in cells concomitantly exposed to 10 nM Exe-4.

Alongside the main goal of the present work, let us point out that, thanks to the proposed approach based on fast and robust spatiotemporal correlation spectroscopy, the structural and dynamic properties of ISGs from living primary human β-cells is readily extracted and can be compared to other β-cell-like standards. Despite the undeniable usefulness of immortalized models, in fact, it is useful to understand how much these are affordable/predictive models as compared to their primary, human-derived counterparts. Here, for instance, the *i*MSD analysis revealed that the granule fingerprint in human-derived β-cells is substantially different from that of immortalized INS-1 E cells, as previously measured by some of us [8].

We envision several lines of development based on present results. From a methodological point of view, the *i*MSD method proved to be a fast and robust approach to screen the structural and dynamic properties of subcellular structures, such as the ISG, in different experimental conditions. It requires only a microscope equipped for fast acquisition, and the structure of interest can be tagged to any genetically encoded or organic fluorophore, thus enabling also multi-channel imaging. Related to this, we believe that cross-*i*MSD analysis will be used in the near future to select sub-populations of subcellular structures to probe their interaction and co-diffusion within the cell.

A few limitations shall be discussed: first, by using *i*MSD, the information on single objects (e.g., trajectories) is inevitably lost as quantitative parameters are extracted as average over the entire population of diffusing objects captured by imaging. This, in turn, implies that intracellular heterogeneity of both the structural and dynamic properties of the object of interest is averaged out. Finally, any detail related to the large amount of molecular information enclosed in dynamic subcellular nanostructures is averaged out during the measurement due to poor temporal resolution. Theoretically, however, no technical limit is present to the possibility to retrieve molecular information, provided that sufficient acquisition speed can be achieved [42].

From a biomedical point of view, the natural prosecution of the present study entails the use of intact islets, where inter-cellular signals/feedbacks may play an important regulatory role that in disaggregated cells is inevitably lost. Moreover, the use of disaggregated cells implies that β-cells are cultivated on 2D supports (e.g., glass) and not maintained in the natural 3D context of the tissue, and this in turn inevitably induces cell morphological rearrangements with potential effects on functional cell properties. To apply the present approach to intact islets, however, a few technical and methodological issues have to be addressed, the main being that proper fluorescence labeling shall be achieved in the intact islet. This step, in turn, implies the use of either virus-based technologies [43,44] or newly-developed organic dyes for granule labeling [45,46], thus avoiding the potentially perturbative effects of lipofection (i.e., Lipofectamine in the present work) on cells. Among organic dyes, ZIGIR, in particular, shows promising properties as a membrane-permeable, Zinc-chelating agent [45]. As such, it would allow to fluorescently label granules both on 2D-cultured cells and in the intact islet, with the only remaining limitation being that glucagon-containing vesicles will concomitantly become fluorescent within α-cells, as they also contain Zinc ions.

## Figures and Tables

**Table 1:** *i*MSD-extracted parameters of syncollin-EGFP transfected dispersed HI and INS-1E.

Cell	Labelling	Size (nm)	D_m_ (μm^2^/s) × 10^−3^	α	N	SCD *
INS-1 E	Syn-EGFP	333 ± 44	2.1 ± 1.2	0.72 ± 0.14	48	/
dHI	Syn-EGFP	261 ±45	1.0 ± 0.8	0.72 ± 0.17	123	0.536

* SCD, statistical cluster distance: see Cluster Similarity Analysis in Methods Section.

**Table 2:** *i*MSD-extracted parameters for dispersed HI exposed to Ctrl medium, Palm and Palm + Exe.

Condition	[Glucose]	Size (nm)	D_m_ (μm^2^/s) × 10^−3^	A	N
Control	Low	263 ± 49	1.1 ± 0.4	0.70 ± 0.18	44
	High	266 ± 52	2.0 ± 1.1	0.78 ± 0.13	36
Palm	Low	275 ± 50	2.0 ± 1.3	0.72 ± 0.13	28
	High	275 ± 49	2.0 ± 1.6	0.69 ± 0.16	35
Palm + Exe-4	Low	247 ± 32	1.2 ± 0.1	0.70 ± 0.14	29
	High	256 ± 43	2.3 ± 1.0	0.78 ± 0.11	19

## Supplementary Materials

The following are available online at www.mdpi.com/1999-4923/13/9/1403/s1, Figure S1: *i*MSD curves. *i*MSD curves of *n* = 123 analyzed acquisitions of syncollin-EGFP labelled ISGs in HI (green curves) and relative average curve (bold green). In black, average *i*MSD curve of *n* = 48 previously published acquisitions of syncollin-EGFP labelled ISGs in INS-1E cells. Figure S2: Exendin-4 effect on ISGs dynamic. Trajectories (in yellow) of ISGs motion in low glucose control medium (Ctrl—3.3 mM G) compared to granule’s motion after glucose stimulation (16.7 mM G) in Ctrl medium, in case of Palmitate 48 h exposure and in case of Palmitate+Exendin-4 48 h exposure.
