# Supplementary material for: Spatiotemporal Correlation Spectroscopy Reveals a Protective Effect of Peptide-Based GLP-1 Receptor Agonism against Lipotoxicity on Insulin Granule Dynamics in Primary Human β-Cells
Source: Pharmaceutics. 2021 Sep 3;13(9):1403. doi: 10.3390/pharmaceutics13091403 (PMC8464798; doi:10.3390/pharmaceutics13091403)
Supplement: Supplementary file 1 [file pharmaceutics-13-01403-s001.zip › pharmaceutics-1322575-supplementary.pdf]

pharmaceutics

Supplementary Materials: Spatiotemporal Correlation Spectroscopy Reveals a Protective Effect of Peptide-Based GLP-1 Receptor Agonism against Lipotoxicity on Insulin Granule Dynamics in Primary Human β-Cells

Gianmarco Ferri, Marta Tesi, Luca Pesce, Marco Bugliani, Francesca Grano, Margherita Occhipinti, Mara Suleiman, Carmela De Luca, Lorella Marselli, Piero Marchetti and Francesco Cardarelli

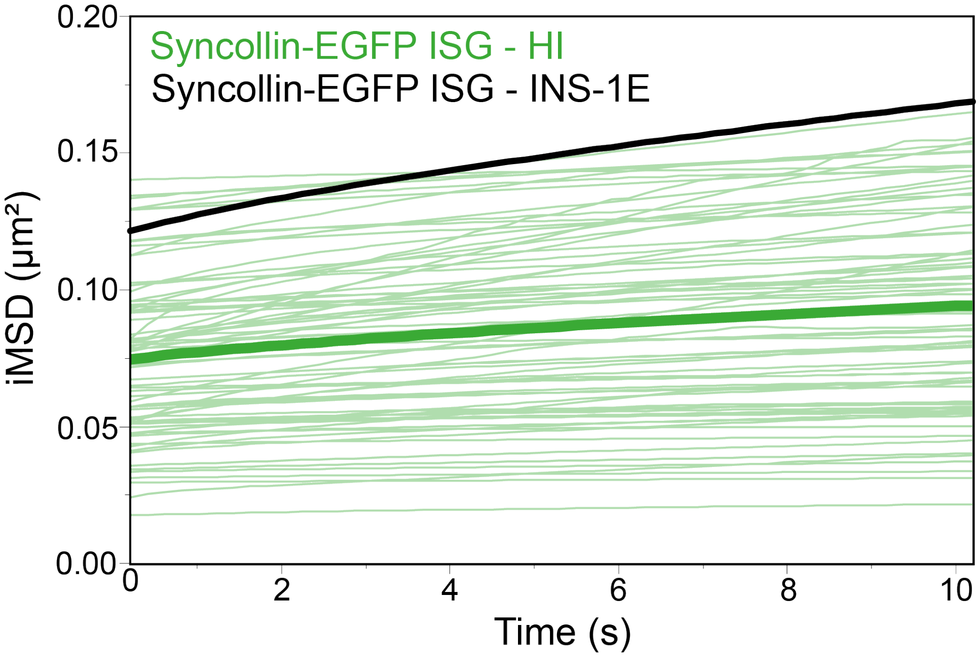

Figure S1. iMSD curves. iMSD curves of $n = 123$ analyzed acquisitions of syncollin-EGFP labelled ISGs in HI (green curves) and relative average curve (bold green). In black, average iMSD curve of $n = 48$ previously published acquisitions of syncollin-EGFP labelled ISGs in INS-1E cells.

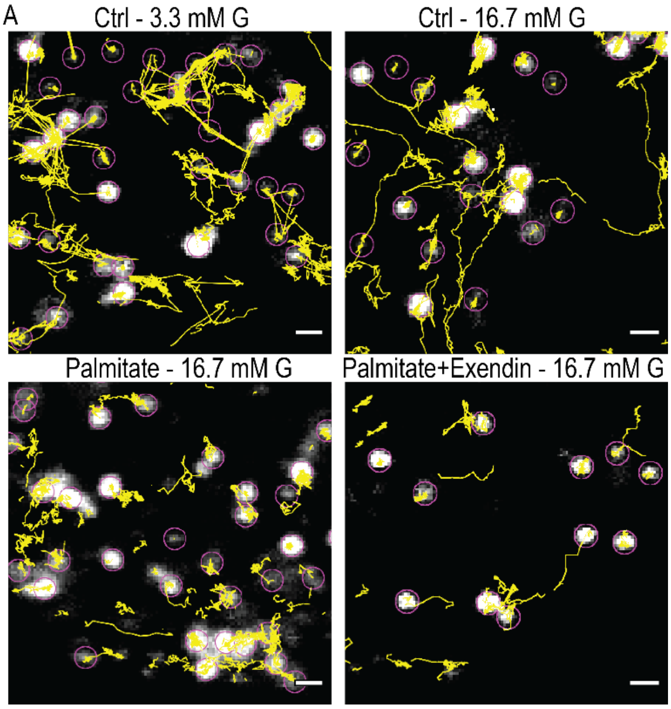

Figure S2. Exendin-4 effect on ISGs dynamic. Trajectories (in yellow) of ISGs motion in low glucose control medium (Ctrl – 3.3 mM G) compared to granule's motion after glucose stimulation (16.7 mM G) in Ctrl medium, in case of Palmitate 48 h exposure and in case of Palmitate+Exendin-4 48 h exposure.
